# Finite element analysis of welded titanium bar and poly ether ether ketone bar in maxillary full arch splinted interim prosthesis

**DOI:** 10.1038/s41598-025-99750-x

**Published:** 2025-05-09

**Authors:** Lamiaa Farouk Zaki Mohamed, Hebatallah Tarek Mohamed, Hany Ibrahim Eid, Soha Saeid Mohammed, Rana Mohammad Abdelrahman

**Affiliations:** 1 Oral and Maxillofacial Prosthodontics Department, Faculty of Dentistry, Ain Shams University, Organization of African Unity Street, Cairo, 11561 Egypt; 2 Oral and Maxillofacial Prosthodontics Department, School of Dentistry, Newgiza University, Giza, Egypt

**Keywords:** Medical research, Materials science

## Abstract

Full arch fixed provisional restorations are prone to fracture during function for several reasons. Those types of fractures during the healing period eliminate the cross-arch stabilization and disrupt stress distribution patterns. FEA (Finite Element Analysis) study was carried out using edentulous maxillary models where implants and other components were represented in three dimensional (3D) geometric models. Two 3D FEA models with six implants were used. Model TB: the implants were splinted with welded titanium bar; Model PB: the implants were splinted with PEEK (Poly Ether Ether Ketone) bar. An interim full arch PMMA (Poly methyl methacrylate) prosthesis was virtually designed for both models. Both models were subjected to vertical and oblique forces with a single force magnitude of 100 N. The amount of maximum equivalent Von-Mises stresses was calculated at the cervical part of the bone cylinder (marginal bone) and both frameworks. Under bilateral vertical loading, stresses were found to be comparable at the marginal bone between titanium and PEEK splinting. The PEEK framework had better and lower stress distribution than the titanium. While under unilateral oblique forces PEEK had better mechanical response on the marginal bone. And PEEK framework itself showed higher stresses than the titanium. The behavior of PEEK and Titanium splints are comparable under the vertical bilateral load. On the contrary to the oblique load, where the stresses are higher within the PEEK splint that correspondingly transmit less stresses to the underlying structures. So, PEEK was found successful in regards to the pattern of stress distribution to both implants and marginal bone, but further studies are needed to confirm its effectiveness and broader applicability.

## Introduction

Rehabilitation of edentulous patients with an implant-supported prosthesis typically involves the placement of implants and then, loading the prosthesis after 3–4 months in the mandible and 6–8 months in the maxilla^1^. Such a stress-free healing period permits the osseointegration of the implant, lowering the risk of implant failure and loss^2^. Two-stage protocol showed significant psychological and functional difficulties for patients who have recently extracted their remaining teeth never worn a removable prosthesis, since they must wear an interim removable prosthesis. Accordingly, the original two-stage protocol has been altered where implants were placed after immediate tooth extraction along with the immediate placement of a fixed interim prosthesis^3^.

Interim prostheses have several advantages, including serving as a diagnostic tool, helping evaluation of peri-implant soft tissue, evaluation of neighboring teeth, and evaluation of the patient’s oral hygiene. They also are helpful for patient management (esthetic, phonetic, psychological), communication between patient, prosthodontist, and technician, determination for implant site development, healing of the soft tissue around the implants, potentially loading the implants, improving tissue contours related to emergence profile, and developing of an interdental or papillae, and therefore potential avoidance of a third surgical procedure^4,5^.

According to Lin et al., the most common complications associated with an interim full-arch fixed acrylic resin prosthesis are the fracture of the prosthetic structure and fracture of the veneering material^6^. Such fracture during the healing period eliminates cross- arch stabilization and disrupts stress distribution patterns^7^.

Several reasons have made the full arch fixed Interim prostheses susceptible to fracture during function, acrylic porosities and/or foreign materials embedded in the acrylic as “Air pockets” have been observed in the cracked acrylic bases of many restorations^8^. It was reported that flexural fatigue, which occurred after repeated flexing of the PMMA prosthesis, could also lead to fractures^9^.

Splinting (rigid or non-rigid) has been shown to prevent axial rotation and movement of immediately loaded implants. However, the effectiveness of splinting to promote implant osseointegration was found to be uncertain since previous studies have shown osseointegration of un-splinted immediately loaded implants^10^. According to some studies, splinting of implants reduces the risk of overload to each implant because of the increased surface area and enhanced biomechanical distribution^11,12^.

Splinting could be either rigid by a metal bar or semi-rigid with acrylic resin. The semi-rigid splinting can be fabricated by CAD/CAM technology at the correct vertical dimension of occlusion before an immediate load, or the patient’s pre-existing denture can be used after adjusting it to serve as a fixed interim prosthesis^13^. Semi-rigid splinting doesn’t provide adequate stress reduction on the bone surrounding the implants. On the other hand, it offers better passivity to the implant- supported fixed prosthesis because of the lack of a metal support. Accordingly, semi-rigid splinting has been recommended for the healing period after implant placement (4–6 months), which is important for implant survival^14^.

Several studies reported successful oral rehabilitation of edentulous maxilla and mandible with a fixed definitive restoration supported by an intra-orally welded titanium wire on the same day of implant placement surgery^15^. The intra-oral welding technique allows rigid splinting of multiple implants for immediate loading on the same day, thus ensuring a predictable fixation of implants in the healing period with a considerable reduction in the micro-movements^16^.

Intraoral welding was reported to be a technique sensitive procedure. It is crucial to have total contact between the welding abutment and the titanium bar during the welding procedure, a perfect joint mandate the application of firm and constant pressure^17^. Furthermore, there are more limits to syncrystallization and electric resistance welding, since they are not efficient on every kind of metal and alloy, and these cannot be utilized on patients with pacemakers^16^.

Polyetheretherketone (PEEK) is a high-performance polymer belonging to the polyaryletherketone group, it has excellent physical and chemical properties as low specific weight (1.3 g/cm^3^) and low flexural strength (165–170 MPa), proper elasticity (3,600 MPa), and suitable hardness (20 HV)^18,19^.

The main beneficial feature of this material is the reduced modulus of elasticity than that of the metallic materials and is relatively comparable to that of the human bone, hence minimizing the stress on the surrounding bone^20^.

In their finite element study to examine the behavior of polyetherketoneketone (PEKK) and polyetheretherketone (PEEK) prosthetic frameworks, Villefort et al.^21^ reported that the superior shock absorbance of PEKK has led to a reduced stress concentration on the prosthetic screw and prosthetic base. This showed clinically a decreased risk of fracture on the acrylic base and screw loosening.

It was reported that the clinical service life of implant prosthesis is influenced by the load transfer between the implant and alveolar bone^22^. Moreover, studies on traditional implants have demonstrated that stress concentration is prevalent in the marginal cortical bone corresponding to the neck of the implant^23^. This stress overload maximizes the risk of marginal bone resorption around the implant^24^. Since marginal bone quality is a critical parameter controlling the osteo-mechanical behavior in implant biomechanics^25^.

Finite element model is a computer technique for stress distribution analysis that has been used for making virtual models. The influence of loading strengths over peri-implant and dental implant elements can be recorded by applying the equivalent stress (von Mises stress) expressed in Megapascals (MPa)^26^.

The finite element analysis (FEA) study has significant clinical implications, as it offers a helpful understanding of the biomechanical behavior of the splinting materials. Additionally, it would help in predicting the reaction of these materials under various loading conditions^27^. Consequently, improving the long-term clinical stability of the implant system and the overall performance.

Moreover, FEA has been shown to be an applicable method for analyzing the distribution of stress and clinical behavior of geometrically complex prosthetic systems that include dental implant, bone, and prosthesis^28^, accordingly, clinicians will be able to identify areas of high stresses that may cause bone loss or future implant failure and therefore, they can follow better planning and maintenance protocols^21^.

This can definitely improve the clinical decision-making, the treatment outcomes, hence reduce the risk of complications. So, the applications of theses FEA studies can guarantee a safe and effective real clinical applications.

Even with all these benefits, FEA studies comprise a number of limitations. The loads tend to be more simplified than the actual complex forces encountered during mastication and various other functions^29^, in addition, they can’t fully capture the nature of the anatomical structures as the bone or the soft tissues. Bone is an incredibly complicated living structure and its characteristics vary from individual to individual. In addition, the use of FEM when studying the extremely accurate anatomy of a bone structure may limit the analysis to that particular structure^30^.

Moreover, FEA is not time-dependent. Biological dynamics of a living component cannot be modeled with precision as it is difficult to take factors like time and other factors the structure is subjected to in accounts. And to obtain a high quality FEA, a specialized software is needed which may not be available to all researches^31^.

Thus, the purpose of this study was to evaluate the mechanical behavior of maxillary full arch loaded interim prostheses fabricated using different splinting materials. The null hypothesis was that the use of different splinting materials would not affect the prosthesis’ mechanical behavior.

## Methods

The current study included two steps: virtual model construction and three-dimensional Finite Element Analysis. Two virtual models were made in this study. Both with six implants are used. In model TB, the implants were splinted with welded titanium bar; however, in model PB, the implants were splinted with PEEK bar.

### Model construction

**Fig. 1:**
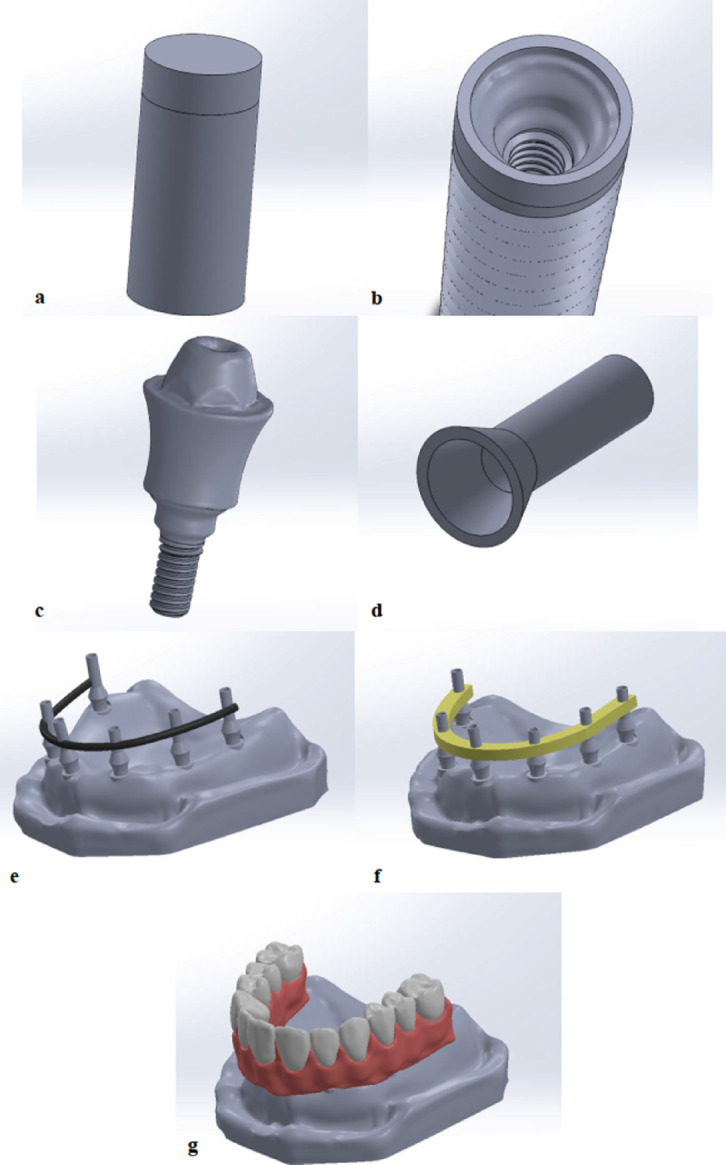
Model designing; (**a**) bone cylinder, (**b**) implant, (**c**) multi-unit abutment, (**d**) temporary titanium sleeve, (**e**) implants splinted with titanium bar of 2 mm thickness titanium wire, (**f**) Implants splinted with PEEK bar of 3 mm thickness, (**g**) prosthetic superstructure designed using Exocad software to be in the form of full arch PMMA prosthesis. Image courtesy of Dr. Nermeen Ahmed Hassan, published under a CC BY open access license with permission.

For virtual model construcAmerication, an educational maxillary edentulous cast (Ramses medical products, Cairo, Egypt) was used. It was scanned using 3D scanner (CeraMap 400 Amann Girrbach Inc., Koblach, Austria) and modelled using Exocad software (Exocad DentalDB 3.1 Rijeka CAD/CAM software) (Exocad America, Inc. Darmstadt, Germany). As (D3) bone density is often observed in the maxilla, the cast was virtually formed to represent a 1 mm outer cortical bone covering the trabecular bone (Fig. 1a). Reverse Engineering was made to the cast and exported in Standard Tessellation Language (STL) file. This STL virtual cast was imported into the Mesh Mixer software (Version 3.5.0) (Mesh Mixer, Autodesk, San Rafael, California, United States of America) for further smoothening, gap filling and exported as STL format^32^.

For the bone cylinder, a new plane was created parallel to the top plane, and a split was made in the top part of the cylinder along the new plane to facilitate collecting the results from the marginal peri-implant bone. Therefore, the marginal peri-implant bone is 2 mm height and 1.3 mm thickness^33^.

Six dental implants were planned to be placed in the right lateral incisor R2, right first premolar R4, right first molar R6, left central incisor L1, left canine L3, left first molar L6. All implants were placed axially^34^.

The implants were modelled using the Solidworks software (2023 SP0 software) (Solidworks 2023 × 64 Edition premium package) with 4.2 mm diameter and a 10 mm length^35–37^ (Fig. 1b). Straight Multiunit abutments and titanium bases were also modelled using the Solidworks software (Fig. 1c and d).

In model TB, the implants were splinted with titanium bar of 2mm thickness^16^ (Fig. 1e) while in model PB, the implants were splinted with PEEK bar of 3mm thickness^21,38^ (Fig. 1f). All other components of both models were identical to standardize all the variables.

The prosthetic superstructure was designed using Exocad software (Exocad DentalDB 3.1 Rijeka CAD/CAM software) (Exocad America, Inc. Darmstadt, Germany) to be in the form of full arch PMMA prosthesis^39^ (Fig. 1g). The STL files of the dental implants, abutments and prosthetic superstructure were assembled by Solidworks software.

Straight multiunit abutment with 5 mm diameter was exported as STL file from BlueSky Plan version 4.12.13 (64 bit) (Bio software, LLC). The STL file was then exported to Solidworks and was reversed engineered to form a 3D cast model^32^.

For the titanium sleeves, a 2D sketch of circle with 5 mm diameter was drawn in the top plane of the cap part. This circle was extruded using boss extrude tool to form a cylinder of 8 mm length. Finally, a cavity was made inside the cap part and the cylindrical part to form the final 3D hollow Titanium sleeve^32^.

The two models were exported to ANSYS software (Ansys 2022 R1) (Ansys, Inc, Pennsylvania, USA) to start the analysis process, the cervical part of each bone cylinder was assumed to be compact bone and the remaining part of the bone cylinder was assumed to be cancellous bone. The implant, multi-unit abutment, temporary titanium sleeves and the metal wire are all assumed to be made from titanium alloy. The PEEK framework was assigned to be made from BioHPP PEEK. While all the conversion prosthesis was assumed to be made from PMMA. The materials were assumed to have linear isotropic behavior and the modulus of elasticity and Poisson’s ratio for the different component materials used in the study are listed in the table below, Table 1. All components were constructed in a way that assures 100% contact along the interfaces.

**Table 1:** Component properties.

Material	Modulus of elasticity (MPa)	Poisson’s ratio
Compact bone	15,000	0.3
Cancellous bone	1500	0.3
PMMA	2770	0.35
Titanium Alloy	110,000	0.3
BioHPP PEEK	4200	0.38

MPa Mega Pascal unit.

Properties of each component in the model.

BioHPP, high performance polymer; PMMA, polymethyl methacrylate.

**Fig. 2:**
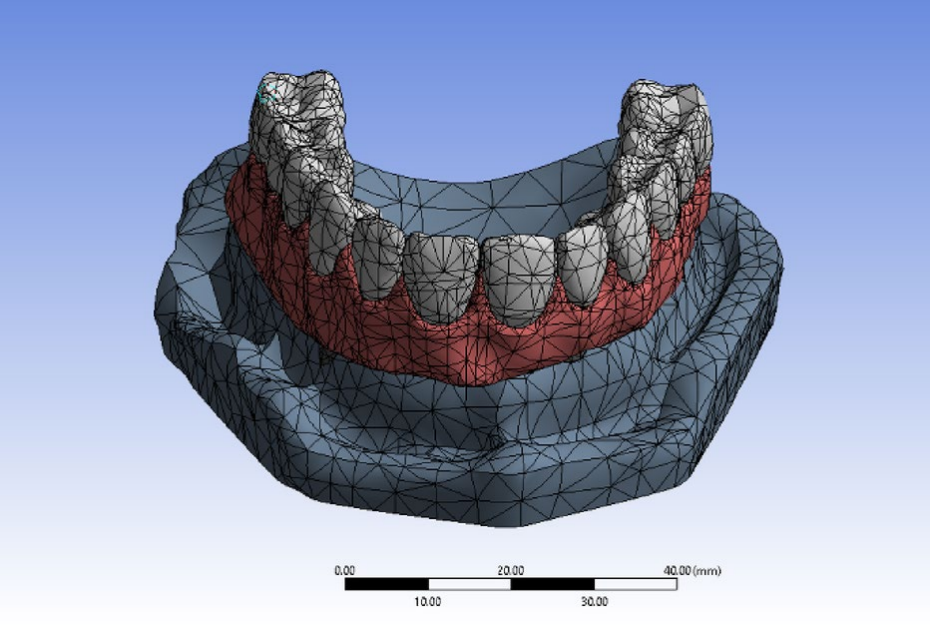
Model meshing. Image courtesy of Dr. Nermeen Ahmed Hassan, published under a CC BY open access license with permission.

Meshing the models were done by subdividing the geometric model into small pieces called elements connected at common points called nodes. (Fig. 2).

The total number of elements in the model TB was 139,733 and in the model PB was 147,951. However, the number of nodes in the model TB was 139,733 and in the model PB was 268,653.The fixed restraints were applied to the inferior aspect and to all the lateral aspects of the maxillary virtual cast to avoid any bodily displacement during loading.

### Load application

**Fig. 3:**
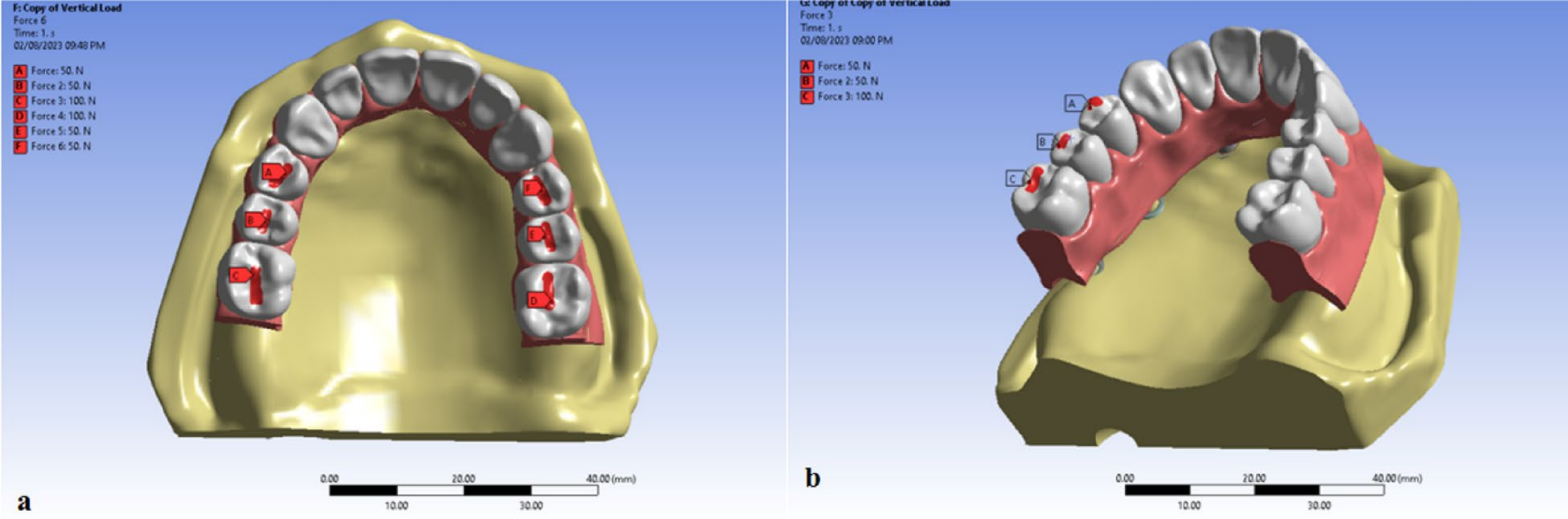
Load application; (**a**) application of bilateral vertical load of 200 on the central fossae premolar and the first molar (**b**) application of unilateral oblique load to the lingual inclines of the buccal cusps of the premolar and first molar with an angle of about 45 degrees to the vertical axis of the tooth. Image courtesy of Dr. Nermeen Ahmed Hassan, published under a CC BY open access license with permission.

Two loading scenarios were applied^33,40,41^. The first scenario simulated a Vertical Load of 200 N that was applied bilaterally in the posterior region where the first molar tooth received load of 100 N and each premolar received 50 N load (Fig. 3a).

The vertical load was applied on the central fossae of the premolar and the first molar. The second scenario simulated an Oblique (Lateral) Load of 200 N was applied unilaterally in the posterior region of the right side where The first molar tooth received load of 100 N and each premolar received 50 N load (Fig. 3b). Lateral load was applied to the lingual inclines of the buccal cusps of the premolar and first molar with an angle of about 45 degrees to the vertical axis of the tooth.

**Fig. 4:**
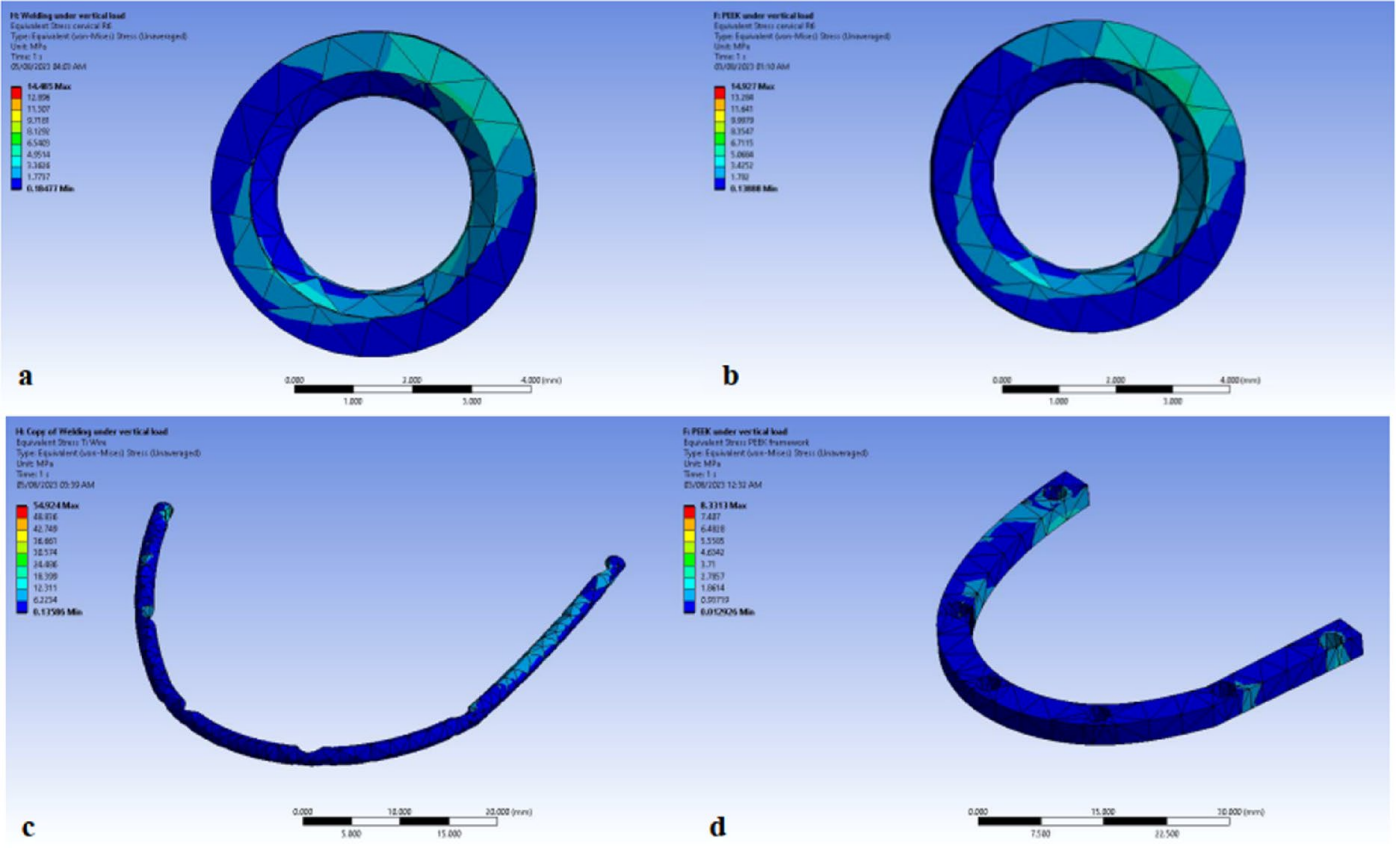
Stress distribution during the application of bilateral vertical load on (**a**) Marginal bone in Titanium model(TB), (**b**) Marginal bone in PEEK model(PB), (**c**) titanium wire, (**d**) PEEK framework. Image courtesy of Dr. Nermeen Ahmed Hassan, published under a CC BY open access license with permission.

**Fig. 5:**
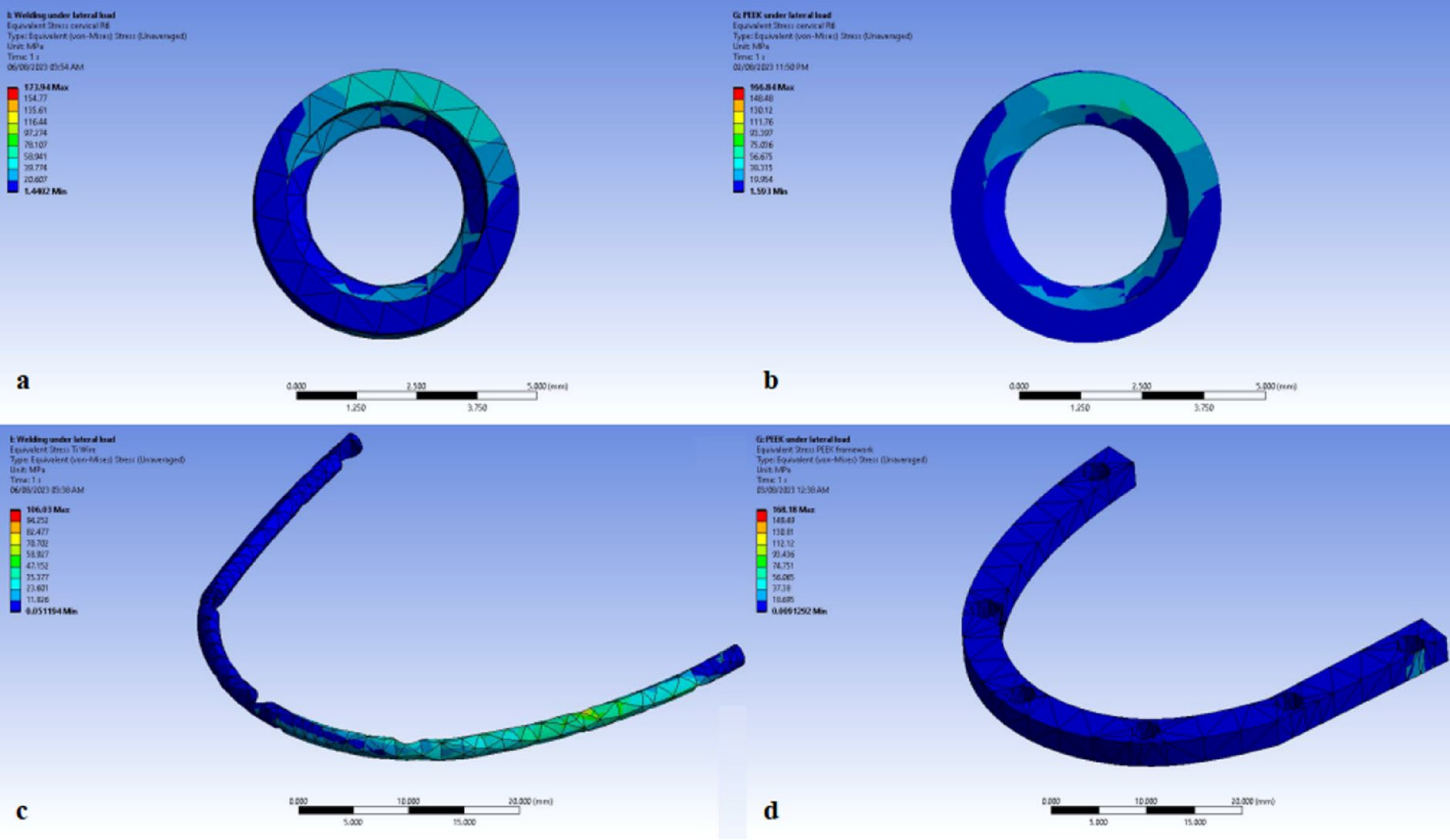
Stress distribution during the application of unilateral oblique load on (**a**) marginal bone in titanium model (TB), (**b**) marginal bone in PEEK model (PB), (**c**) titanium wire, (**d**) PEEK framework. Image courtesy of Dr. Nermeen Ahmed Hassan, published under a CC BY open access license with permission.

In both models, the stresses displayed are the maximum principle stress for the (1) cervical part of the bone cylinder (marginal bone) (Figs. 4a and b, 5a and b). and (2) titanium wire (Figs. 4c and 5c). and PEEK framework (Figs. 4d and 5d).

**Fig. 6:**
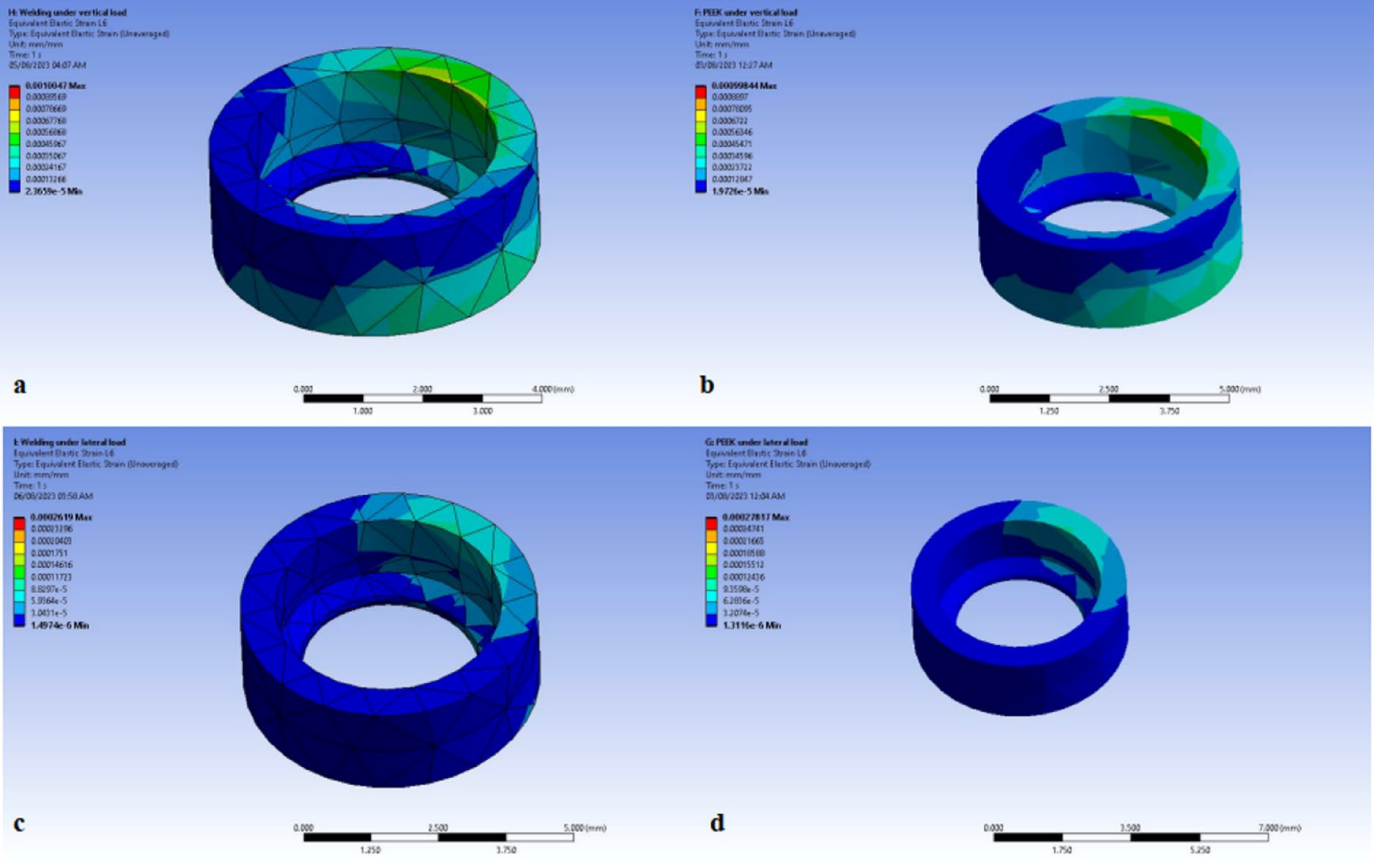
Strain distribution during the application of bilateral vertical load on (**a**) marginal bone in titanium model (TB), (**b**) marginal bone in PEEK model (PB), strain distribution during the application of unilateral oblique load on (**c**) marginal bone in titanium model (TB), (**d**) marginal bone in PEEK model. Image courtesy of Dr. Nermeen Ahmed Hassan, published under a CC BY open access license with permission.

In both models, the strain displayed for the (1) cervical part of the bone cylinder (marginal bone) under vertical load (Fig. 6a and b). and (2) cervical part of the bone cylinder (marginal bone) under oblique load (Fig. 6c and d).

They were displayed as graphical output in the form of color coded maps and numeric output that displayed the amount of the maximum equivalent stresses (Von-Mises stresses) in Megapascal (Mpa)^39,40^. Then, the results were collected from both models and tabulated. 

## Results

The results of this study showed that under bilateral vertical load, the marginal bone stresses at the left and right molars were 15.07 and14.485 MPa receptively for Model TB (titanium framework), and 14.977 and 14.927 MPa receptively for Model PB (PEEK framework). The results recorded were comparable in both models.

Moreover, it was found that under bilateral vertical load, PEEK framework had better distribution and lower stresses than titanium framework. The stresses were 8.3313 and 54.924 MPa in PEEK and titanium frameworks respectively Table 2. This may be contributed to the connection between PEEK bar and abutments. Regarding the stresses on the implants, it was found that the results in Model TB are slightly higher than that in Model PB, and this may be contributed to the shock absorbing nature of PEEK that tends to dissipate stresses under the bilateral vertical load which accordingly transmits less stresses to the implants.

**Table 2:** Equivalent (von-Mises) stresses in case of bilateral vertical load.

Bilateral vertical loading												
	Peek						Titanium					
Marginal bone	Left first molar	Left canine	Left central	Right lateral	Right first pre-molar	Right first molar	Left first molar	Left canine	Left central	Right lateral	Right first pre-molar	Right first molar
	14.9	17.5	4.5	4.1	13.3	14.9	15.1	18.5	5.6	4.7	13.3	14.4
Framework	8.3						54.9					

Comparison of the maximum recorded stress values in the marginal bone and the framework between the models PB and TB during the bilateral vertical loading scenario.

PEEK, polyetheretherketone.

Under unilateral oblique forces, the marginal bone stresses at the left and right molars were 3.9284 and 173.94 MPa receptively for Model TB. Whereas for Model PB, they were found to be 4.1725 and 166.84 MPa respectively. PEEK is showing better mechanical response compared to titanium framework. While PEEK framework showed higher stresses than the titanium framework. The stresses were 168.18 and 106.03 MPa respectively. And again, this may be contributed to the nature of the connection between PEEK bar and abutments Table 3. In regards to the stresses on the implants, it was found again that the results in Model TB are higher than that in Model PB, and this may be caused by the nature of PEEK under the oblique load that tends to accumulate the stresses within the PEEK itself transmitting less stresses to the implants.

**Table 3:** Equivalent (von-Mises) stresses in case of unilateral oblique load.

Unilateral oblique load												
	Peek						Titanium					
Marginal bone	Left first molar	Left canine	Left central	Right lateral	Right first pre-molar	Right first molar	Left first molar	Left canine	Left central	Right lateral	Right first pre-molar	Right first molar
	4.1	11.1	37.1	46.7	126.4	166.8	3.9	16.6	43.5	51	128.9	173.9
Framework	168.1						106					

Comparison of the maximum recorded stress values in the marginal bone and the framework between the models PB and TB during the unilateral oblique loading scenario.

PEEK, polyetheretherketone.

Regarding the strain, it was found that that under bilateral vertical load, the marginal bone strain at the left and right molars were 8.90 and 9.66 mm/mm MPa receptively for Model TB (titanium framework), 9.98 and 9.95 mm/mm receptively for Model PB (PEEK framework). The results recorded were comparable in both models (Table 4).

**Table 4:** Equivalent elastic strain (mm/mm) in case of bilateral vertical load.

Bilateral vertical loading												
	PEEK						Titanium					
Marginal bone	Left first molar	Left canine	Left central	Right lateral	Right first pre-molar	Right first molar	Left first molar	Left canine	Left central	Right lateral	Right first pre-molar	Right first molar
	9.98 mm/mm	1.17 mm/mm	3.06 mm/mm	2.71 mm/mm	8.9 mm/mm	9.95 mm/mm	8.90 mm/mm	1.24 mm/mm	3.74 mm/mm	3.18 mm/mm	8.88 mm/mm	9.66 mm/mm

Comparison of the maximum recorded strain values in the marginal bone between the models PB and TB during the bilateral vertical loading scenario.

PEEK, polyetheretherketone.

While under unilateral oblique forces, the marginal bone strain at the left and right molars were 2.621 and 1.166 mm/mm receptively for Model TB. Whereas for Model PB, they were found to be 2.78 and 1,11 mm/mm respectively. The results recorded were comparable in both models (Table 5).

**Table 5:** Equivalent elastic strain (mm/mm) in case of unilateral oblique load.

Unilateral oblique load												
	PEEK						Titanium					
Marginal bone	Left first molar	Left canine	Left central	Right lateral	Right first pre-molar	Right first molar	Left first molar	Left canine	Left central	Right lateral	Right first pre-molar	Right first molar
	2.78 mm/mm	1.38 mm/mm	2.48 mm/mm	3.12 mm/mm	8.43 mm/mm	1.11 mm/mm	2.62 mm/mm	1.11 mm/mm	2.90 mm/mm	3.40 mm/mm	8.60 mm/mm	1.16 mm/mm

Comparison of the maximum recorded strain values in the marginal bone between the models PB and TB during the unilateral oblique loading scenario.

PEEK, polyetheretherketone.

## Discussion

The present study evaluated the stress distribution along the marginal bone using two different splinting materials under vertical and oblique loads. The results showed that both splinting materials have different mechanical responses, thus, the null hypothesis was rejected.

The clinical evaluation of the stress and strain distribution in peri-implant bone areas is difficult and not feasible in most situations^.^ Thus, FEA is an appropriate method for evaluating the distribution of stresses and clinical performance of geometrically complex prosthetic systems that include dental implant, bone, and prosthesis^28^.

As the study that ran by Altıparmak et al., where three—dimensional finite element analysis was used to examine the stresses that occur under occlusal forces on the bone, and the implant systems made of Titanium and Polyether ether ketone (PEEK) material^42^.

Since FEA is a simulation, increasing the mesh density in the regions to be evaluated on the model improved the data accuracy. Like previous finite element analysis studies related to implantology research, our study assumed that all modeled structures are in constant contact with each other^43^.

Regarding the influence of the number of implants on the structure, it is proved that the placement of six implants distribute stresses more evenly. In a study conducted by Fazi et al.^44^ the stress distribution in 3–4–5 implant-supported prosthesis was investigated and it was found that load accumulation on the system declined with 5 implant-supported prosthesis.

In a recent study by Desai et al.^40^, two loading forces were applied, bilateral vertical forces which represented a clenching situation, and the unilateral which simulated the masticatory forces.

In order to simulate the mean posterior bite force values, a vertical load of 200 N (50 N for each premolar and 100 N for the first molar) was applied on the central fossae of the premolars and the first molar. On the other hand, the oblique load was applied to the lingual inclines of the buccal cusps of the premolars and the first molar with an angle of about 45 degrees to the vertical axis. This is consistent with the studies done by Osman et al., and Hassan et al.^33,41^.

Also, Meric et al.^45^, showed that materials used in the fabrication of dental prostheses can influence the loading of dental implants and may potentially lead to bone deformation.

Assunção et al.^46^, concluded that the use of rigid framework materials in implant supported prostheses increased the amount of stress on dental implants. Unlike the study conducted by Dayan et al.^47^, which concluded that the fabrication of frameworks from rigid materials in All-on-4 prostheses reduces stress in dental implants and peri-implant bone when the distal implants are tilted 30°.

Other studies, as the one performed by Çiftçi and Canay^48^, and the study ran by Sertgöz^49^, where they reported that the use of less rigid restorative materials resulted in high stress in dental implants and supporting tissues.

Garg ^17^, demonstrated that rigid framework splinting obtained by intraoral welding, can control the micro-movements in immediate loading protocols and decrease the mechanical stresses exerted on each single implant, accordingly, assuring an optimal distribution of occlusal load and reducing the lateral forces on healing implant.

Although, some studies as the one performed by Aboelnagga M, where it was concluded that using polyether-ketone-ketone (PEKK) for constructing the framework of prosthesis led to favorable stress distribution and reduction of stresses induced to supporting implants^50^.

Also, El-Rahman et al.^51^, studied the behavior of PEEK bar under cyclic loading, where it exerted less strain and showed favorable load distribution around the implants and on the ridge areas.

The data of this study showed that the intensity and distribution of the equivalent stresses, in unilateral oblique loads, in the PEEK framework were higher than the Ti frameworks. While under bilateral vertical load the intensity and distribution of the equivalent stresses were higher in Ti frameworks. This might give a speculation of an improved load distribution along the PEEK framework in model PB compared to model TB. Such a speculation might be related to the shock absorption and low elastic modulus of PEEK.

The findings of this study coincide with the study done by Ersöz and Mumcu^52^, and Jaros et al.^53^, which showed that PEEK responded differently under oblique and axial load; as under oblique loading PEEK accumulated stresses instead of distributing it, in contrast to the stiffer CoCr, ZrO2 and Ti. This is based on the material’s ability for shock absorption and the low elastic modulus.

Also, the study done by Elkhooly AS et al., examined the pattern of stress distribution along PEKK and Bio-HPP CAD-CAM bar materials in implant-supported prostheses. They found that under unilateral loads, the strains concentrated at the loaded implants and ridge leading to statistically significantly larger mean micro-strains on the loaded side compared to the unloaded side^54^.

In accordance to our study, Yu et al.^55^ reported that compared to polymeric frameworks, zirconia and metals caused lower stresses on bone and implant, and greater stresses on framework.

It was reported that the biomechanical qualities of high elastic modulus materials made them more appropriate for implant-supported prostheses frameworks^56^. A three-dimensional finite element analysis study done by Shin et al.^57^, concluded that the framework with the low elastic modulus (PEKK) reduced the stress within the framework; however, it transmitted additional stress to the supra-structures of the prostheses and this was consistent with the finding of this study.

Similarly, Tribst et al.^58^ concluded that an increase in the elastic modulus of the framework decreased the stress transmitted to the implants and surrounding bone.

In the study done by Shetty et al.^59^ where full-arch implant supported prosthesis was evaluated with different frameworks by strain gauge analysis, PEEK demonstrated higher deformation values than ZrO2 and CoCr in the presence of cantilever which coincide with the finding of this study.

In contrast to our study, Franco et al.^60^ reported that the cross section of the PEEK splint has its effect on the response to the applied stresses. However, in this study the cross section effect wasn’t evaluated.

Regarding the clinical relevance, and based on the current study results, the biomechanical properties and behavior of the PEEK splint qualifies it to be used with the provisional restoration, keeping in mind that PEEK response to oblique load tends to accumulate rather than dispense the stresses along the splint body, so the usage of cantilever is not advised in such a case to avoid the risk of bending or even fracture of the splint. Additionally, the design of the implant platform, occlusal forces pattern, implant number and diameter have their effect on the pattern of the stress transmission^61^.

Though standardization and variables control can be obtained from finite element analysis studies. Yet, this study has many limitations. Static loading was applied for the purpose of simplification whereas loading is dynamic during the normal chewing functions. Moreover, it was proposed that all dental implants were fully Osseointegrated with bone, however, this does not mimic the clinical situation. And, the material properties of the bone were proposed to be linearly elastic and isotropic, however, this does not coincide with the living tissue simulation^32^.

## Conclusion

Within the limitations of our study and the outcomes obtained, it was concluded that the behavior of PEEK and Titanium splints are comparable under the vertical bilateral load. On the contrary to the oblique load, where the stresses are higher within the PEEK splint that correspondingly transmit less stresses to the underlying structures. So, PEEK was found successful in regards to the pattern of stress distribution to both implants and marginal bone, but further studies are needed to confirm its effectiveness and broader applicability

## Data Availability

All data generated or analyzed during this study are provided within the manuscript.

## Abbreviations

STL: Standard tessellation language
N: Newton
MPa: Mega Pascal
PEEK: Polyetheretherketone
